# Common-path interferometer for digital holographic Doppler spectroscopy of living biological tissues

**DOI:** 10.1117/1.JBO.26.3.030501

**Published:** 2021-03-29

**Authors:** Kwan Jeong, Maria Josef Lopera, John J. Turek, David D. Nolte

**Affiliations:** a Korea Military Academy, Department of Physics, Seoul, Republic of Korea; b Universidad EAFIT, Department of Physics, Medellín, Colombia; c Purdue University, Department of Basic Medical Sciences, West Lafayette, United States; d Purdue University, Department of Physics, West Lafayette, United States

**Keywords:** digital holography, biological imaging, spectroscopy, interferometry, optical coherence tomography, dynamic contrast

## Abstract

**Significance:** Common-path interferometers have the advantage of producing ultrastable interferometric fringes compared with conventional interferometers, such as Michelson or Mach–Zehnder that are sensitive to environmental instabilities. Isolating interferometric measurements from mechanical disturbances is important in biodynamic imaging because Doppler spectroscopy of intracellular dynamics requires extreme stability for phase-sensitive interferometric detection to capture fluctuation frequencies down to 10 mHz.

**Aim:** The aim of this study was to demonstrate that Doppler spectra produced from a common-path interferometer using a grating and a spatial filter (SF) are comparable to, and more stable than, spectra from conventional biodynamic imaging.

**Approach:** A common-path interferometer using a holographic diffraction grating and an SF was employed with a low-coherence source. Simulations evaluated the spatial resolution. DLD-1 (human colon adenocarcinoma) spheroids were used as living target tissue samples. Power spectra under external vibrations and drug-response spectrograms were compared between common-path and Fourier-domain holographic systems.

**Results:** The common-path holography configuration shows enhanced interferometric stability against mechanical vibrations through common-mode rejection while maintaining sensitivity to Doppler frequency fluctuations caused by intracellular motions.

**Conclusions:** A common-path interferometer using a grating and an SF can provide enhanced interferometric stability in tissue-dynamics spectroscopy for drug screening assays.

Tissue-dynamics spectroscopy (TDS) is a fluctuation spectroscopy based on Doppler light scattering from intracellular dynamics^1^^,^^2^ that tracks changes in intracellular motions in response to applied xenobiotics or cancer therapeutics.^3^^,^^4^ The motion sensitivity required for TDS applications also requires extreme mechanical stability for phase-sensitive measurements of Doppler frequency shifts down to 10 mHz. Previous TDS systems used a Mach–Zehnder off-axis holographic configuration that enabled *en face* optical coherence tomography.^5^ However, dual-arm interferometers such as Michelson and Mach–Zehnder interferometers are subject to environmental influences such as mechanical vibrations and thermal drifts.

Common-path interferometers^6^^–^^11^ have been widely applied in a variety of applications, including optical coherence tomography^12^^,^^13^ and digital holography,^14^^–^^19^ due to their inherent insensitivity to vibrations. In a common-path configuration, the object and reference waves share the same optical path from the sample interaction volume to the detector. A variety of configurations using a 4-f image system and a diffraction grating at the Fourier plane have been developed.^20^ In this letter, we propose and demonstrate a common-path interferometer using a grating and a spatial filter (SF) to enable stable TDS of living thick biological tissue for drug screening assays.

The common-path interferometric configuration is developed for reflective mode operation as shown in Fig. 1. A Superlum S840-B-I-20 superluminescent diode, with a center wavelength at 840 nm and a short coherence length of 10  μm, is used as the light source. The probe beam illuminates the target at an oblique angle of 34 deg relative to the backscatter direction. Intracellular transport is isotropic relative to the direction of the incoming wave vector, and the average Doppler frequency shift is zero. The knee frequency of the fluctuation spectrum represents the maximum Doppler frequency shift within the ensemble of scatterers, and the maximum is at the backscattering angle of 180 deg, but we use the slight angled illumination on the sample to eliminate the beam splitter and increase the intensity of the collected light. A 10× microscope objective lens with a long working distance of 30.5 mm and a numerical aperture of 0.26 is employed to collect the light scattered from the target. After relaying the light by lenses L1 and L2 at a magnification of 3:2, a holographic transmission grating (G) at the first Fourier plane (FP1) splits the light into identical +1 and −1 diffraction orders. The phase grating (HOLO/OR LTD) has a 73% transmission efficiency (with a partially quenched zero-order) and a beam separation angle of 2.01 deg. The light is transformed by lens L3 to a second image plane (IP2) where only the first orders pass through the SF. The other diffraction orders are blocked by the SF consisting of a small reference aperture of 0.6 mm diameter and a large object aperture of 3.0 mm in diameter. The two apertures are separated laterally by 5.3 mm. The +1 diffractive order through the large aperture is the object wave, whereas the −1 diffraction order through the small aperture produces the reference wave. An optical Fourier transform is performed by the Fourier lens L4, and the reference and object waves share the same path to create stable interference fringes at the second Fourier plane (FP2). The hologram with a size of 800 by 800 pixels is recorded by a CMOS camera (Basler acA1920-155um) with 12-bit depth at 25 frames per second and an exposure time of 10 ms.

**Fig. 1:**
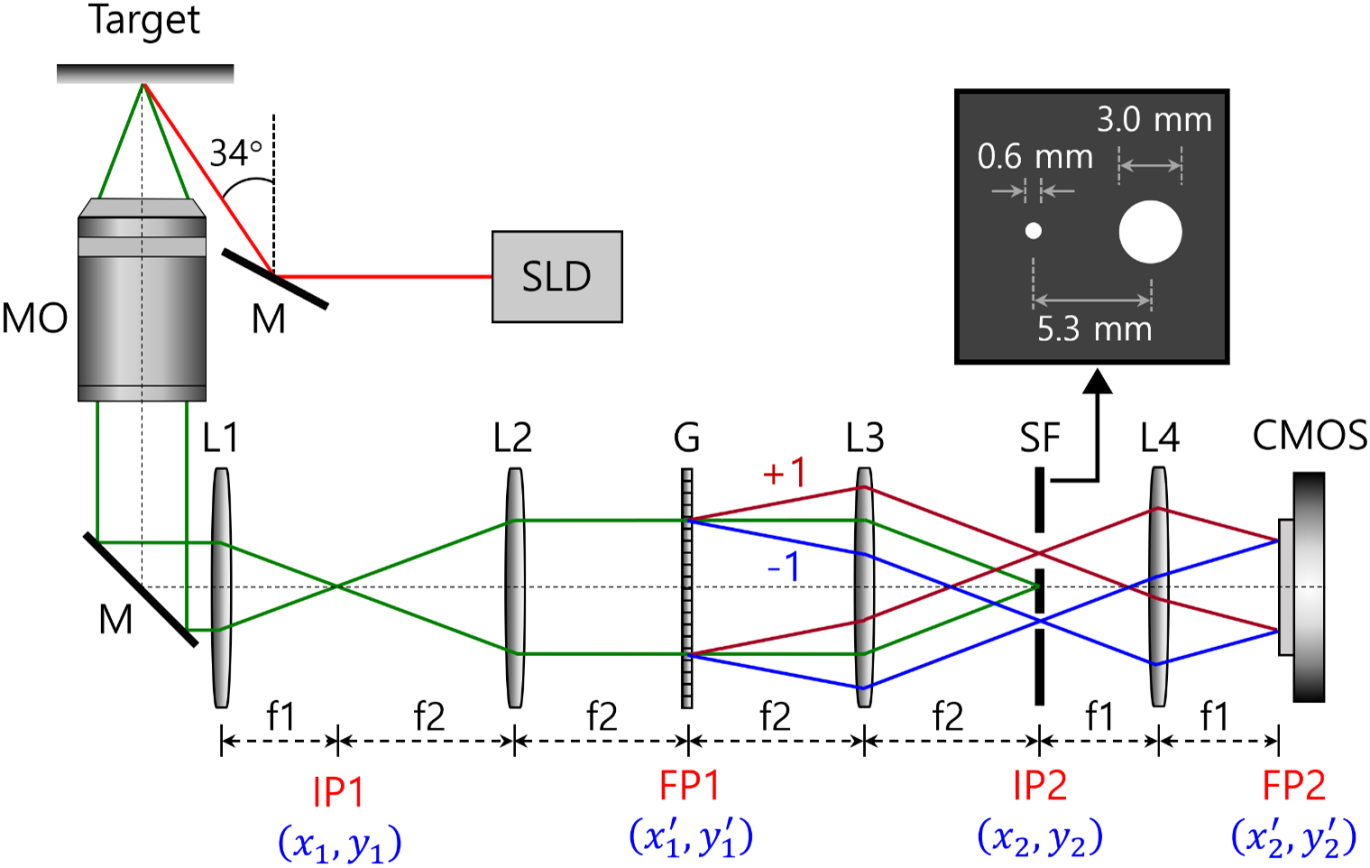
Experimental set-up of CDH system. MO, microscope objective; M, mirror; FPn, Fourier plane n; IPn, image plane n; Ln, lens n; G, grating; SF, spatial filter, f1=10  cm, f2=15  cm.

The scattered wave field magnified by the microscope objective MO and the lens L1 is denoted as $U(x_{1},y_{1})$ at the image plane IP1. For an ideal binary phase grating with a duty cycle of 0.5 and a π-phase depth, the transmission function of the grating is given as

$$g(x_{1}^{\prime },y_{1}^{\prime })=\overset{+\infty }{\underset{m=-\infty }{\sum }}\frac{\sin(m\pi /2)}{m\pi /2}\;\exp[j\frac{2\pi mx_{1}^{\prime }}{\Lambda }],$$ (1)

where m is the diffraction order and Λ is the period of the grating. The diffraction efficiency vanishes for all even values of m and has a maximum of about 40.5% for each of the +1 and −1 orders. In the 4f imaging system of the lenses L2 and L3, the wave $U_{a}(x_{2},y_{2})$ after the SF at IP2 is given as

$$U_{a}(x_{2},y_{2})\approx U(-x_{2}+x_{0},-y_{2})\mathrm{circ}[r_{+}^{\prime }/R_{o}]+U(-x_{2}-x_{0},-y_{2})\mathrm{circ}[r_{-}^{\prime }/R_{r}],$$ (2)

where $x_{0}=\lambda f_{2}/\Lambda$, λ is the wavelength, $r_{+}^{\prime }=\sqrt{{\left(x_{2}-x_{0}\right)}^{2}+y_{2}^{2}}$, $r_{-}^{\prime }=\sqrt{{\left(x_{2}+x_{0}\right)}^{2}+y_{2}^{2}}$, and $R_{o}$ and $R_{r}$ are the radii of the object and reference aperture, which are centered at $+x_{0}$ and $-x_{0}$ in the x direction from the center, respectively.

The last lens L4 performs the Fourier transform, and the reference wave $U_{r}(x_{2}^{\prime },y_{2}^{\prime })$ and object wave $U_{o}(x_{2}^{\prime },y_{2}^{\prime })$ at the camera plane FP2 can be expressed as

$$U_{o}(x_{2}^{\prime },y_{2}^{\prime })\approx \exp(j\frac{2\pi x_{0}x_{2}^{\prime }}{\lambda f_{1}})FT[U]⊗J_{\mathrm{inc}}(\frac{R_{o}\rho^{\prime }}{\lambda f_{1}}),$$ (3)

$$U_{r}(x_{2}^{\prime },y_{2}^{\prime })\approx \exp(-j\frac{2\pi x_{0}x_{2}^{\prime }}{\lambda f_{1}})FT[U]⊗J_{\mathrm{inc}}(\frac{R_{r}\rho^{\prime }}{\lambda f_{1}}),$$ (4)

where FT[U] is the Fourier transform of U, $J_{\mathrm{inc}}(x)=J_{1}(x)/x$ in which $J_{1}(x)$ is a Bessel function of the first kind, ⊗ denotes convolution, and $\rho^{\prime }=\sqrt{{\left(x_{2}^{\prime }\right)}^{2}+{\left(y_{2}^{\prime }\right)}^{2}}$. The reconstruction of the hologram is performed by an inverse Fourier transform and consists of a zero-order term and two conjugate diffraction terms. One diffraction term is reconstructed at $\left(2x_{0},0\right)$ as

$$FT^{-1}\langle \exp(j\frac{4\pi x_{0}x_{2}^{\prime }}{\lambda f_{1}}){\left\{FT[U(\frac{-x_{2}^{\prime }}{\lambda f_{1}},\frac{-y_{2}^{\prime }}{\lambda f_{1}})]⊗J_{\mathrm{inc}}(\frac{R_{r}\rho^{\prime }}{\lambda f_{1}})\right\}}^{*}\;\times \{FT[U(\frac{-x_{2}^{\prime }}{\lambda f_{1}},\frac{-y_{2}^{\prime }}{\lambda f_{1}})]⊗J_{\mathrm{inc}}(\frac{R_{o}\rho^{\prime }}{\lambda f_{1}})\}\rangle .$$ (5)

In common-path digital holography (CDH), the resolution and phase sensitivity of the reconstructed image are determined by the reference-wave term of $FT[U]⊗J_{inc}(R_{r}\rho^{\prime }/\lambda f_{1})$ in Eq. (4). In Fourier-domain digital holography (FDH),^21^ on the other hand, this term is replaced by the plane reference wave and the quality of the reconstructed image is mainly determined by the object wave FT[U]. Therefore, the spatial resolution in FDH is higher than that for CDH.

The digital holograms recorded at the camera plane are numerically transformed using a two-dimensional fast Fourier transform (FFT). An example of target image reconstructions using CDH is shown in Fig. 2(a), where the target is matte white paper containing an oblique cut-out line. The figure shows images of the reference and object beams for the two types of reference aperture at the image plane IP2, and their reconstructed images are shown after the FFT. As shown in Eq. (5), the first-order image when using the small reference aperture with a diameter of 0.6 mm is similar to the target image at IP2, whereas the first-order image for the reference aperture with the same diameter as the object aperture is the autocorrelation of the object. Therefore, it is necessary to optimize the size of the reference aperture for biodynamic imaging to maximize the throughput while maintaining acceptable resolution for biodynamic imaging applications.

**Fig. 2:**
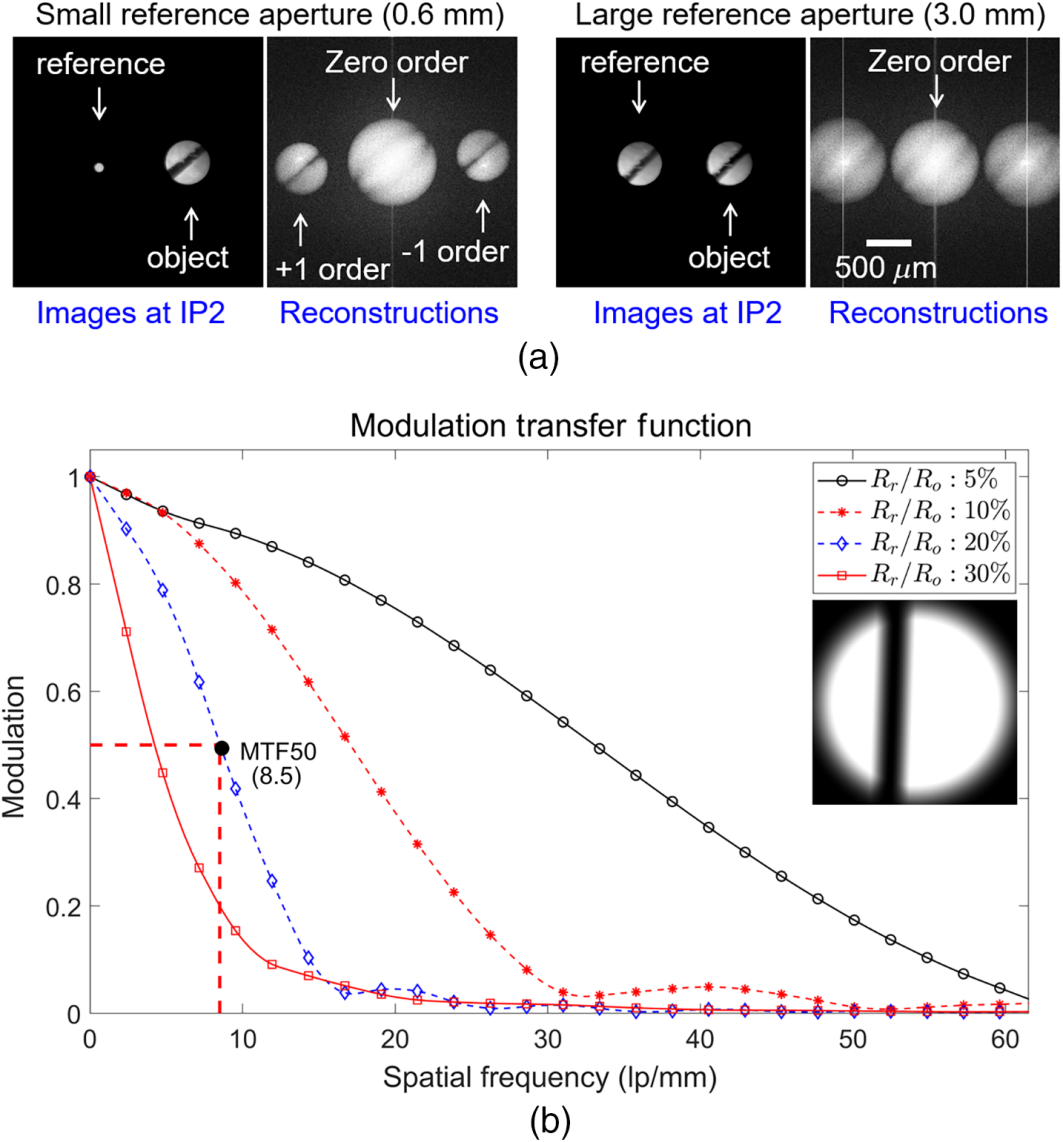
(a) Images for the two types of reference aperture at the IP2 image plane and their reconstructed images. (b) MTFs for four different ratios of $R_{r}/R_{o}$ to estimate the resolution of the CDH system. Inset: simulated reconstruction of a slanted-edge phase target with a π-phase depth for $R_{r}/R_{o}=20\%$.

Simulations were performed using Eq. (5) to estimate the optimal size of the reference aperture by measuring the modulation transfer function (MTF). The MTF of an imaging system is defined as the output modulation, $M_{o}$, divided by the input modulation, $M_{i}$, as a function of spatial frequency. A vertical knife-edge phase target with a π-phase depth and a tilt angle of 2 deg is used as the object image for the simulation, and the MTFs are evaluated from the Fourier transform of the line spread functions^22^ using the reconstructed images obtained by varying the ratio $R_{r}/R_{o}$ from 0.01 to 0.4, as shown in Fig. 2(b).

The reconstructed image for the large reference aperture (100% size ratio) is the autocorrelation of the object image and has no phase information. However, a reference aperture with a smaller diameter at the image plane IP2 acts like a pin hole that makes the reference wave at the FP2 more similar to a plane wave, increasing the spatial resolution, as shown in the graph in Fig. 2(b). However, smaller diameters limit the optical fluence at FP2, which decreases the signal-to-noise ratio and sensitivity in biodynamic imaging. We selected nominal performance using a reference aperture with a 20% ratio relative to the size of the object aperture, and its spatial resolution is estimated to be 8.5 line pairs per mm (lp/mm) in Fig. 2(b). A smaller reference aperture can be used to increase the resolution and phase sensitivity if a source with a higher power or a camera with a higher sensitivity were used. When the reference aperture uses 5% or 10% size ratios, the resolution in Fig. 2(b) can reach 33 or 17  lp/mm, respectively.

We used living DLD-1 (human colon adenocarcinoma) spheroids as a target tissue sample to validate that the CDH system is more stable to external vibrations and more sensitive to biological movements than the noncommon-path FDH system. Tumor spheroids were cultured in a rotating bioreactor for 7–to 14 days until 300 to 600  μm diameter spheroids were formed, and then immobilized with low-gel-temperature agarose in a 96-well plate. To investigate stability against external vibrations, biodynamic imaging of fresh tumor spheroids was performed in both the CDH and FDH modes with and without external vibrations. External vibration was generated by driving a motor with adjustable coupling to the optical table. The speed of the motor was increased until it noticeably affected the interference fringes observed in the hologram at about 6000 rpm. Holograms were recorded for 82 s at a frame rate of 25 frames per second while imaging a fresh tumor spheroid of about 300  μm diameter. Cross-sections (lateral position versus time) in FDH and CDH modes with external vibrations are shown in Fig. 3(a). In the cross-section of FDH in the conventional Mach–Zehnder configuration, the total intensity fluctuates over time caused by the table vibrations, whereas in the common-path CDH, the intensity at each pixel only fluctuates over time due to intracellular motility, and the total intensity is stable.

**Fig. 3:**
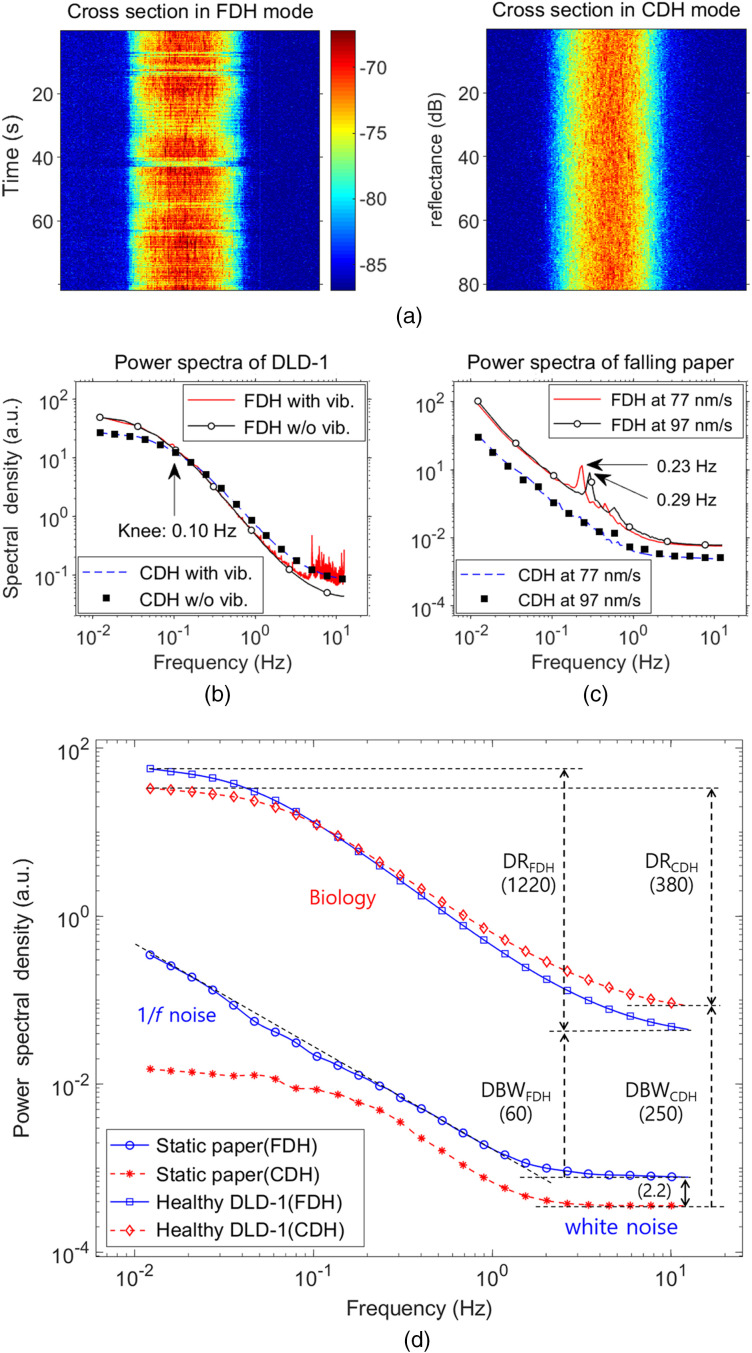
(a) DLD-1 living tumor spheroid cross section as a function of position (horizontal axis) and time (vertical axis) in FDH and CDH modes with external vibrations. (b) Power spectra corresponding to (a) compared to those without external vibrations. (c) Power spectra of slowly falling paper. (d) Power spectra of dynamic sample (DLD-1) and static sample (white paper) in FDH and CDH.

Fluctuation power spectra are acquired by performing temporal FFTs of the time series of multiple reconstructions performed at the specified frame rate as shown in Fig. 3(b). The line plots without markers are the power spectra corresponding to Fig. 3(a), and the plots with markers are for those with no vibration, demonstrating that the CDH system is more stable to external vibration than the FDH system. To compare the effects of external vibrations quantitatively, the relative deviation at a frequency is defined as the absolute difference between the value of the spectral density with and without vibration divided by the value of the spectral density with no vibration. The averages of relative deviations in three bands (lower than 0.1 Hz, between 0.1 and 1 Hz, higher than 1 Hz) were measured to be 4.6%, 3.1%, and 80%, respectively for FDH, whereas 1.8%, 2.5%, and 2.4%, respectively, for CDH when using this high-frequency vibration source. The power spectrum in FDH has considerable noise, especially in the high frequency band, whereas the power spectrum in CDH is relatively smooth for all frequency bands. This curve shows the typical shape of living tissue,^23^ with a knee at low frequency (0.1 Hz), a power-law roll-off at intermediate frequency, and a floor near the Nyquist frequency. To simulate low-frequency noise, a slowly falling paper target (caused by paper floating of the surface of a water-filled well as the water evaporates) is used as a slow phase modulation source. As shown in Fig. 3(c), the FDH power spectra show peaks at 0.23 and 0.29 Hz for two different paper speeds, but no peaks in the CDH mode. This illustrates the insensitivity of CDH to global phase drift because of common-mode rejection, which is the central reason why it is stable against external mechanical disturbances. Figure 3(d) compares the power spectrum of a dynamic DLD-1 spheroid with the power spectrum of static white paper in FDH and CDH at roughly the same backscatter brightness. Compared to the spectral density of static paper, the white noise in FDH is 2.2 times higher than that in CDH due to the difference in stability against external vibrations. The detection bandwidth in CDH is 4.2 times higher than that in FDH, whereas the dynamic range (DR) of biological samples in FDH is 3.2 times higher than that in CDH due to the more efficient photon collection in FDH. Therefore, the CDH system is insensitive to global phase drift while remaining sensitive to speckle-scale phase fluctuations caused by interfering Doppler frequency shifts from intracellular motions.

To validate that the CDH system is an effective modality for TDS, tissue-response spectrograms tracking time changes in intracellular dynamics in response to drug perturbations were acquired in both FDH and CDH modes as shown in Fig. 4. To make this comparison, the FDH system was operated with maximum vibration isolation to achieve the highest stability relative to CDH. Spectrograms are generated from spectral changes relative to the average baseline spectrum in fluctuation power spectra as a function of time. After six measurements of predose baselines, postdose responses capture the signatures caused by the treatment and are measured 15 times every hour. Cytoskeletal (nocodazole and paclitaxel) and metabolic (iodoacetate) drugs were used, and medium containing 0.1% carrier dimethyl sulfoxide (DMSO) (for drug solubility) was used as a negative control. Nocodazole disrupts microtubules by inhibiting the polymerization of microtubules, whereas paclitaxel stabilizes microtubules by preventing depolymerization. Iodoacetate inhibits the glycolysis that contributes to the rapid growth of cancer cells through the supply of ATP.

**Fig. 4:**
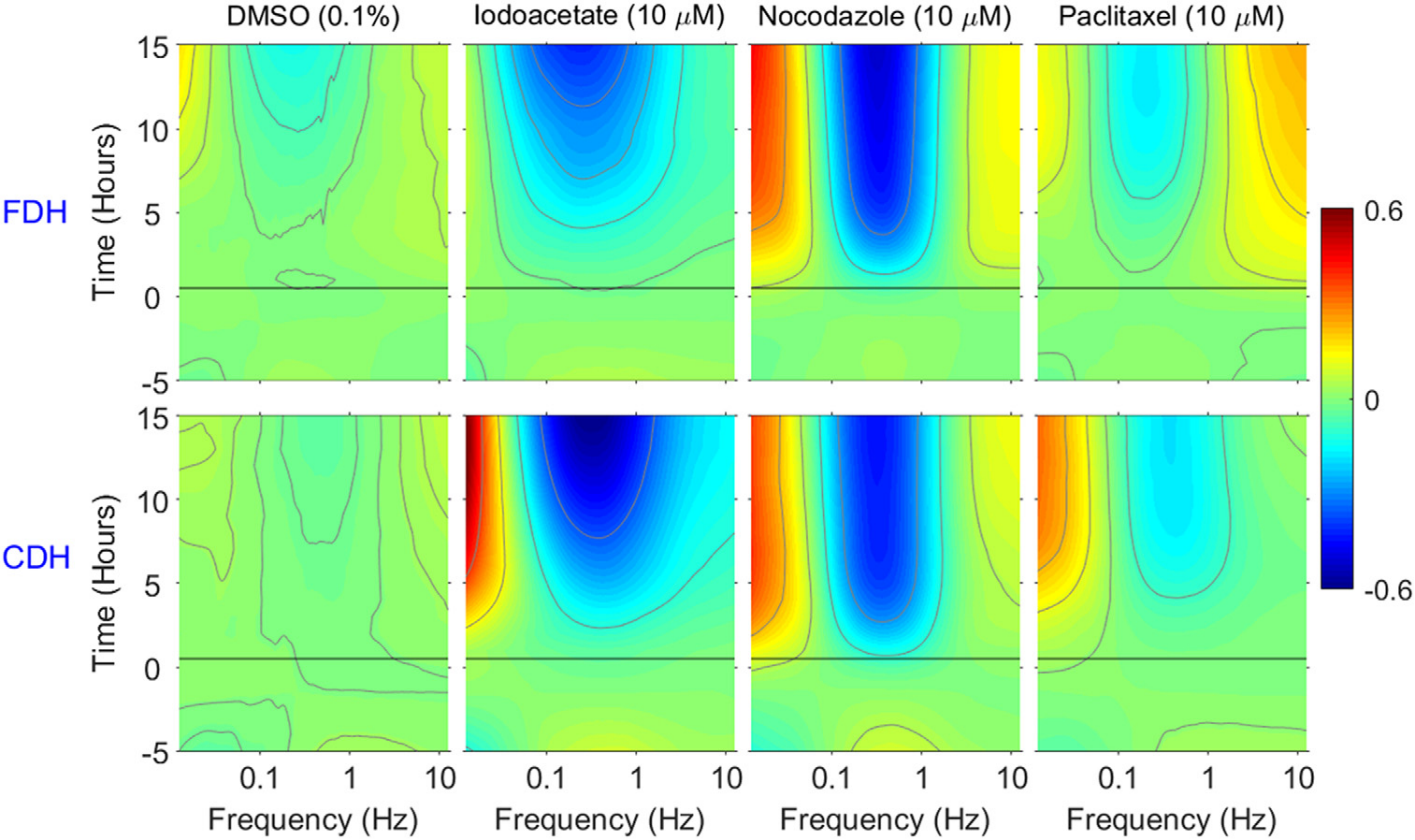
Average spectrograms (three replicates) showing the drug-responses to 0.1% DMSO, iodoacetate, nocodazole, and paclitaxel in FDH and CDH. The drug was applied at t=0. The CDH performance is functionally equivalent to FDH.

Spectrograms for the negative control in FDH and CDH modes show relatively small response typically seen in continuously proliferating tissue samples, with minor changes over time. The average spectrograms in response to iodoacetate in FDH mode indicates an overall inhibition of cellular activity. In CDH mode, the tissue-response is close to that of FDH mode except for the stronger redshift in CDH at low frequencies. The response to nocodazole showed enhanced low and high frequencies and suppression at mid-frequencies with minor differences between the FDH and CDH modes. The response to paclitaxel closely matches that of nocodazole, except for a weaker response. Therefore, tissue responses to drugs are functionally equivalent between the CDH and FDH modes, whereas the CDH system has the added advantage of stability against environmental influences and may have more flexibility than the FDH system in point-of-care applications despite its lower resolution.

Achieving enhanced interferometric stability using this common-path holography configuration required several trade-offs on biodynamic performance. For instance, the common-path system trades off spatial resolution against holographic fringe contrast on the camera. For the application of imaging tumor spheroids, nominal performance is achieved by limiting the reference aperture to 20% of the image aperture that limits the imaging resolution to ∼100  μm. This resolution, though insufficient to image individual cells in the tumor, is compatible with the tissue dynamics spectroscopic imaging mode of biodynamic imaging,^24^ which produces spatial maps of intracellular dynamics across the tumor. Another trade-off is the loss of independent z-control for depth ranging. On the other hand, the low-coherence light source produces a condition of self-coherence-gating that selectively interferes light that shares the same optical path length. This creates a “compressed flythrough” that superposes successive coherence-gated holograms, at increasing depth, onto the digital camera. The Doppler spectra are averaged over all depths of the target weighted by a decreasing exponential function that decays with the reduced extinction coefficient $\mu^{\prime }$. This selectively weights the Doppler information to a depth of ∼200  μm inside the target with the lateral spatial resolution of 100  μm discussed above. Biodynamic imaging into thick samples with moderate amounts of multiple scattering increases dynamic sensitivity because Doppler frequency shifts accumulate with each scattering event and increases the sensitivity to intracellular motions. Therefore, the 20-μm voxel size of conventional biodynamic imaging is traded for ∼100-μm voxel size in this common-path system while gaining superior mechanical stability and maintaining full spectral DR for tissue dynamics applications. Measuring changes in intracellular motion in living tissues to extract functional information is a growing area in optical coherence tomography.^25^^–^^28^
